# Decoy receptor 3: an endogenous immunomodulator in cancer growth and inflammatory reactions

**DOI:** 10.1186/s12929-017-0347-7

**Published:** 2017-06-19

**Authors:** Shie-Liang Hsieh, Wan-Wan Lin

**Affiliations:** 1 Genomics Research Center, Academia Sinica, 128 Academia Road, Section 2, Nankang, Taipei, 115 Taiwan; 2 Institute of Clinical Medicine & Immunology Research Center, National Yang-Ming University, Taipei, Taiwan; 3 Department of Medical Research and Education, Taipei Veterans General Hospital, Taipei, Taiwan; 4 Institute of Immunology, College of Medicine, National Taiwan University Taipei, Taipei, Taiwan; 5 Institute for Cancer Biology and Drug Discovery, Taipei Medical University, Taipei, Taiwan; 6 Department of Pharmacology, College of Medicine, National Taiwan University, No. 1 Section 1, Jen Ai Road, Taipei, 10001 Taiwan

**Keywords:** Decoy receptor 3 (DcR3), M2 macrophages, Biomarker, TNFR6B

## Abstract

Decoy receptor 3 (DcR3), also known as tumor necrosis factor receptor (TNFR) superfamily member 6b (TNFRSF6B), is a soluble decoy receptor which can neutralize the biological functions of three members of tumor necrosis factor superfamily (TNFSF): Fas ligand (FasL), LIGHT, and TL1A. In addition to ‘decoy’ function, recombinant DcR3.Fc is able to modulate the activation and differentiation of dendritic cells (DCs) and macrophages via ‘non-decoy’ action. DcR3-treated DCs skew T cell differentiation into Th2 phenotype, while DcR3-treated macrophages behave M2 phenotype. DcR3 is upregulated in various cancer cells and several inflammatory tissues, and is regarded as a potential biomarker to predict inflammatory disease progression and cancer metastasis. However, whether DcR3 is a pathogenic factor or a suppressor to attenuate inflammatory reactions, has not been discussed comprehensively yet. Because mouse genome does not have DcR3, it is not feasible to investigate its physiological functions by gene-knockout approach. However, DcR3-mediated effects in vitro are determined via overexpressing DcR3 or addition of recombinant DcR3.Fc fusion protein. Moreover, CD68-driven DcR3 transgenic mice are used to investigate DcR3-mediated systemic effects in vivo. Upregulation of DcR3 during inflammatory reactions exerts negative-feedback to suppress inflammation, while tumor cells hijack DcR3 to prevent apoptosis and promote tumor growth and invasion. Thus, ‘switch-on’ of DcR3 expression may be feasible for the treatment of inflammatory diseases and enhance tissue repairing, while ‘switch-off’ of DcR3 expression can enhance tumor apoptosis and suppress tumor growth in vivo.

## Background

DcR3 has been shown to be a pleiotropic soluble factor to modulate cell functions via ‘decoy’ and ‘non-decoy’ actions [1]. DcR3 is able to inhibit apoptosis and enhance angiogenesis via neutralizing members of TNF superfamily FasL [2, 3], LIGHT [4], and TL1A [5, 6]. In addition, DcR3 also skews macrophages into M2 phenotype [7, 8] and promotes tissue repairing [9] via the ‘non-decoy’ action. Because the TL1A/DR3 (TL1A receptor) axis has been reported as a novel immune pathway that participates in the pathogenesis of a variety of autoimmune rheumatic diseases, DcR3 may become a promising therapeutic target for inflammatory arthritis [10].

DcR3 expression is hardly detectable in physiological condition. Thus, most of studies focus on DcR3 expression in pathological conditions, and verify DcR3-mediated effects in DcR3 transgenic mice which overexpress DcR3 locally [11] or systemically [3]. The basic feature of DcR3 has been reviewed extensively [1], however, the information regarding the physiological functions of DcR3 is relatively limited. In this review article, we discuss the potential effector functions of DcR3 in maintaining homeostasis. Inhibition of DcR3 expression may attenuate tumor growth, while enhancement of DcR3-mediated effector functions may become a promising approach to attenuate autoimmunity and promote tissue repairing. Thus, recombinant DcR3 is a promising therapeutic agent for immunotherapy, while switching off DcR3 expression in cancer microenvironment may enhance the efficacy of cancer therapy.

## Decoy receptor 3 (DcR3) and its ligands

Decoy receptor 3 (DcR3) is a member of the tumor necrosis factor receptor (TNFR) superfamily member 6b (TNFRSF6B)/TR6/M68. DcR3 cDNA is initially identified from human cancer cells, and DcR3 gene amplification is also found in certain cancer cell lineages [2]. Unlike most members of TNFRSF, DcR3 lacks a transmembrane domain, and is detectable in serum and cell culture medium. DcR3 is able to bind and neutralize the functions of three members of the tumor necrosis factor superfamily (TNFSF): FasL (CD95L/TNFSF6) [2], LIGHT (CD258/TNFSF14) [4], and TNF-like molecule 1A (TL1A/VEGI/TNFSF15) [5].

The TL1A/death receptor 3 (DR3/TNFRSF25) axis is a novel immune pathway that participates in the pathogenesis of a variety of autoimmune rheumatic diseases [10], thus molecules involved in regulating TL1A/DR3 axis are regarded as promising therapeutic targets for various inflammatory and autoimmune diseases [12]. The implication of LIGHT in driving inflammatory and autoimmune diseases such as arthritis, inflammatory bowel disease (IBD), lupus and multiple sclerosis has drawn the attention of many scientists, and blockade of LIGHT-mediated inflammation is now being pursued for the treatment of autoimmune diseases clinically [12].

DcR3 is found in the genome of human (*Homo sapiens*), chimpanzee (*Pan troglodytes*), and most of animals belonging to Phylum Chordata. However, DcR3 does not exist in mouse (*Mus musculus*) and rat (*Rattus norvegicus*) genomes, thus it is not feasible to understand its physiological functions by gene-knockout approach. It has been demonstrated that human DcR3 is able to bind and neutralize the functions of mouse FasL, LIGHT, and TL1A [3]. Thus, transgenic mice overexpressing human DcR3 are applied to investigate DcR3-mediated physiological and pathological effects in vivo.

## Upregulation of DcR3 expression in physiological conditions

Even though DcR3 mRNA and protein are initially identified in cancer cells and various tumor cell lines [2], DcR3 serum level is very low, and almost un-detectable in most of normal individuals not suffering from inflammatory diseases and cancer. Interestingly, DcR3 is detectable in the granulosa cells, endometrial cells, and theca cells [13, 14]. DcR3 is also detectable in undifferentiated primary keratinocytes, and is further upregulated by ultraviolet (UV) light [15], epidermal growth factor (EGF), and transforming growth factor (TGF)-α [16]. Low DcR3 level in serum is detectable during menstrual cycle of women, while DcR3 transcript and protein are upregulated by sex hormones in RL-95 endometrial cells [13]. These observations suggest that DcR3 may play a role in keratinocyte differentiation and egg fertilization.

## Upregulation of DcR3 expression in inflammatory diseases and cancer

It has been shown that DcR3 expression is upregulated in monocytes and dendritic cells (DCs) from patients suffered from silicosis [17] and bacterial infection [18]. Even though DcR3 is detectable in human chondrocytes of osteoarthritis (OA) patients and normal individuals [19], DcR3 concentration in synovial fluids and sera of rheumatoid arthritis (RA) patients is significantly higher than that of OA patients [19]. Moreover, DcR3 mRNA and protein are also found in the fibroblast-like synoviocytes (FLS) of RA [20, 21], but not OA, patients [22]. DcR3 mRNA expression in RA-FLS is induced by IL-23, which plays a critical role in the induction of IL-17 and IL-6-mediated inflammatory disease [23]. Thus, DcR3 level in synovial fluid may become a useful marker to predict the severity of RA.

In addition to cells in the synovial cavity during RA, DcR3 is also upregulated in Kaposi’s sarcoma-associated herpesvirus (KSHV)-infected human umbilical vein endothelial cells (HUVECs) [24] and skin lesions of psoriasis patients [25]. In human keratinocytes, DcR3 is transcriptionally regulated by EGF via NF-κB pathway [16], and contributes to the pathogenesis of psoriasis by impairing the terminal differentiation of keratinocytes (unpublished data). Moreover, enhanced DcR3 expression is found in the majority of idiopathic pulmonary fibrosis (IPF) fibroblasts on collagen matrices [26], as well as in inflamed intestinal mucosa of Crohn’s disease patients [27]. High serum DcR3 levels are reported to associate with occurrence of peritonitis in patients receiving peritoneal dialysis [28, 29].

In addition to inflammatory diseases, DcR3 is upregulated in numerous cancer cells and tumor tissues [1]. DcR3 is detectable in glioma [30], astrocytoma [31], vascular endothelial cells and neighboring lymph nodes of tumor, and its expression level correlates with lymphangiogenesis [32] and lymph node metastasis [33]. DcR3 expression level also correlates positively with clinicopathological change in bladder urothelial carcinoma [34], breast cancer [32], pancreatic head carcinoma [35], colorectal cancer (CRC) [36], gastrointestinal cancer [37, 38], and female reproductive carcinoma [39]. DcR3 upregulation by Epstein-Barr virus (EBV) in SW480 CRC and Burkitt’s lymphoma Akata cell line is via transcription activator Rta [40] and latent membrane protein 1 [41], respectively.

## Serum DcR3 as a biomarker for inflammation and cancer progression

DcR3 in inflammatory region of colon is highly upregulated in patients of IBD [27, 42]. In addition, DcR3 serum level is increased in the patients with primary Sjögren’s syndrome [43], RA [44], and primary biliary cirrhosis [45]. DcR3 level in peripheral blood correlates negatively with the outcome of acute respiratory distress syndrome [46]. Elevation of serum DcR3 level was reported as a risk factor for patients suffered from chronic kidney disease under dialysis [47]. Furthermore, DcR3 serum level correlates with the development of pulmonary arterial hypertension and systemic inflammation in diffuse cutaneous systemic sclerosis patients [48], and is able to predict coronary artery disease severity and major adverse cardiovascular events in patients with multi-vessel coronary artery disease [49]. High serum DcR3 level is also associated with disease severity in non-atopic asthma patients [50]. It is interesting to note the significant positive correlations between the serum DcR3 levels and Birmingham Vasculitis Activity Score (BVAS), myeloperoxidase (MPO), anti-neutrophil cytoplasmic antibody (ANCA) titer, white blood cell counts, serum creatinine levels, and serum C-reactive protein (CRP) levels in ANCA-associated vasculitis (AAV) [51]. Because detection of ANCA is more time consuming and expensive, detection of serum DcR3 by ELISA seems to be an alternative to predict the severity of AAV in the future.

In addition to acute and chronic inflammation, elevation of DcR3 serum level is observed in patients suffered various infectious diseases. Elevated serum DcR3 level was found in patients suffered from sepsis [52], and is a valuable marker to predict the outcome of sepsis [53]. Higher DcR3 serum level correlates with slow progression in HIV-1-infected AIDS patients [54], and is observed in patients with chronic viral hepatitis (CVH) than in controls [55]. Furthermore, DcR3 serum levels is also elevated in decompensated cirrhosis and hepatocellular carcinoma, and is significantly higher compared not only to controls, but also to CVH patients [56].

In cancer patients, serum DcR3 level also correlates with cancer staging. It was reported that malignant plasma cells and T lymphocytes from myeloma patients directly produce DcR3, and serum DcR3 levels in myeloma patients are significantly higher compared to controls [57]. In addition, elevated serum DcR3 is associated with tumor metastasis of oral cavity cancers [58] and bladder transitional cell carcinoma [59]. Recently, serum DcR3 level is not only detected by ELISA, but also by liquid chromatography electrospray ionization mass spectrometry (LC-ESI MS) for absolute quantitation [60]. Thus, it will be feasible to predict the outcome of disease severity by determining the cut-off value of DcR3 serum level in the future.

In cancer and inflammatory diseases, upregulation of DcR3 is via NF-κB activation pathway [41, 61–65], and correlates with ERK expression in gastric cancer [66]. It is interesting to note that treatment of renal cell carcinoma cell lines (ACHN and 769-P) with both the PI3K-inhibitor LY294002 and the AKT-inhibitor IV results in a strongly reduced DcR3 expression on both protein and mRNA levels, indicating DcR3 is upregulated by PI3K/AKT-dependent pathway [61]. Correspondingly, overexpression of the constitutively active form of AKT leads to an increased DcR3 expression [61]. Thus, targeting MEK/ERK and PI3K/AKT pathways in tumor cells may be a novel approach to enhance the efficacy of cancer treatment.

## Functional study of DcR3 in vitro by knockdown or overexpression approaches

Because mouse and rat genomes do not contain DcR3, functional study of DcR3 in vitro is via knockdown of DcR3 expression in human cells by siRNA (loss of function), or overexpression of human DcR3 in mouse cells (gain of function).

Endogenous DcR3 seems able to increase tumor resistance to chemotherapy, because knockdown of DcR3 dramatically increases sensitivity of pancreatic, gastric and ovarian tumor cells to gemcitabine-[67], 5-fluorouracil- [68], and platinum-induced [69] cell death, respectively. Moreover, knockdown of DcR3 by siRNA enhances FasL-induced apoptotic activity, and significantly reduces cell migration and invasion with a decrease in the activation of PI3K/AKT and matrix metalloproteinase (MMP)-2 in human malignant fibrous histiocytoma cells [62]. Furthermore, DcR3 siRNA can enhance pancreatic tumor cells sensitivity to TRAIL-induced apoptosis [70]. Similarly, knockdown of DcR3 in colon cancer cell line SW480 also reduces metastatic activity and the levels of vascular endothelial growth factors (VEGFs) and MMP expression. The DcR3-specific siRNA can efficiently inhibit cell growth and induce apoptosis via attenuating ERK and AKT activation, and the apoptosis rate is increased to 1.85 and 3.93 folds at 72 and 96 h after transfection, respectively [71]. Similarly, knockdown of DcR3 in pancreatic Pata8988 cancer cells reduces ERK1/2 phosphorylation with elevated expression of caspases and increased susceptibility to FasL-induced apoptosis [72]. In addition to siRNA, downregulation of DcR3 by triptolide also triggers the apoptosis of pancreatic cancer cells [67].

In contrast, overexpression of DcR3 promotes adhesion, migration, and invasiveness of tumor cells [61, 62, 73]. The DcR3-dependent tumor invasiveness correlates with the upregulation of urokinase plasminogen activator, MMP7, and integrin α4. DcR3 overexpression significantly enhances tumor proliferation and migration in vitro and tumorigenesis in vivo [73].

## Functional study of DcR3 in vivo by transgenic mice and gene therapy

To understand DcR3-mediated functions in vivo, transgenic mice overexpressing DcR3 by rat-insulin promoter (RIP) [11] and CD68 promoter [3], respectively, were generated to address this question. Overexpression of human DcR3 in islet beta-cells by RIP promoter completely suppresses the onset of insulitis and diabetes in the non-obese diabetes mice [11]. In addition, attenuated Th1 response and increased susceptibility in DcR3-transgenic mice after *Listeria monocytogenes* infection was observed [3]. The attenuation of Th1 response is via the neutralization of TL1A, which induces Th1 and Th17 responses in cooperation with IL-23 [74, 75]. The alternative mechanism is via neutralizing LIGHT, which is critical for IL-12 production and optimal CD4^+^ Th1 responses against parasites [76]. In CD68 promoter-driven DcR3 transgenic (CD68-DcR3 Tg) mice, macrophages display higher levels of IL-10, IL-1RA, Ym1, and arginase activity, whereas the expression of IL-12, TNF-α, IL-6, NO, and MHC class II is downregulated [7, 8]. Furthermore, enhanced tumor growth and spreading are observed in CD68-DcR3 Tg mice, and the enhanced tumor growth is abolished by arginase inhibitor and histone deacetylase inhibitor sodium valproate [8]. This observation provides direct evidence that endogenous human DcR3 has potent effect to skew macrophages differentiation into M2-like phenotype or tumor-associated macrophages (TAMs) via epigenetic regulation [7]. This argument is supported by the fact that DcR3 expression level correlates with M2 macrophage differentiation in papillary thyroid carcinoma [77].

To explore the potential therapeutic effect of DcR3, DcR3 expression plasmids were introduced into mice with autoimmune diseases. It has been shown that administration of CMV promoter-driven human DcR3 plasmid (pCMV-DcR3) into mice can prevent the onset of autoimmune crescentic glomerulonephritis [78]. DcR3 gene therapy also results in improvement of proteinuria, renal function, and renal pathology in a mouse IgA nephropathy model [79].

## Effector functions of DcR3.Fc

**Fig. 1:**
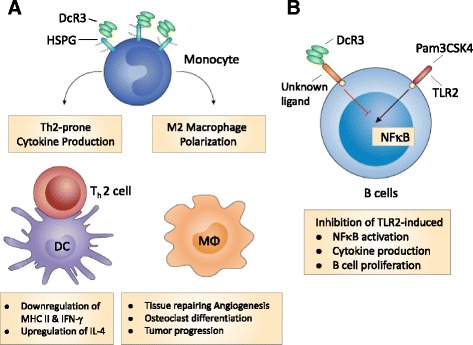
Mechanisms of DcR3.Fc-mediated immunomodulation. **a** DcR3 modulates the activation and differentiation of myeloid cells via HSPGs. DcR3.Fc triggers downstream signaling via syndecan-2 and CD44v3 on monocytes to influence monocyte differentiation into dendritic cells (DCs) and macrophages (Mϕ). DcR3-treated DC skews allogenic T cell differentiation into Th2 phenotype, while DcR3-treated Mϕ displays typical M2 markers, and is able to enhance tumor growth and tissue repairing. **b** DcR3 modulates B cell activation via a yet-identified ligand on B cells. DcR3.Fc suppresses TLR2 ligand (Pam3CSK4) and Staphylococcus aureus cowan (SAC) strain-induced B cell activation via binding non-HSPG ligand(s) on B cells
B. **B cells:**
  DcR3.Fc is able to suppress Staphylococcus aureus Cowan (SAC) strain-induced B cell proliferation as well as TLR2-induced TNF-α secretion [87], NFκB activation, and cytokine (IL-6, TNF-α, IL-12p40, IL-10) secretion via an unknown factor distinct from HSPGs, because DcR3.Fc-mediated effect is not blocked by heparin or heparan sulfate [88]. Unlike myeloid cells, addition of heparin does not inhibit DcR3.Fc binding to B cells (Fig. 1b). As the Fc portion of DcR3.Fc is wild type human IgG1, it would be interesting to test whether DcR3.Fc binds to ITIM-containing Fc receptor (such as FcγR2b) to execute its suppressive effect in the future.
C. **Tumor cells:**
  DcR3.Fc has been shown to bind to ovarian cancer cells via HSPGs, and increase cells resistance to platinum-induced cell death [69]. Compared with wild type CT26 colon cancer cells, enhanced migration and invasion are observed in CT26 cells overexpressing DcR3 (CT26-DcR3). In addition, CT26 cells grow faster and invade to lung tissue after inoculation in CD68-DcR3 transgenic mice than in wild type littermates [8]. This observation suggests that DcR3 and DcR3.Fc can enhance tumor proliferation in vivo (Fig. 2).

Compared with endogenous DcR3, several studies demonstrate that recombinant DcR3.Fc fusion protein is more potent than endogenous DcR3 to modulate cell functions:

A. **Myeloid cells:**
  The recombinant DcR3.Fc fusion protein is able to induce CD14^+^-monocyte differentiation into CD1a^low^CD40^low^CD54^low^CD80^low^CD86^high^ DCs, which then skew T cells differentiation into Th2 phenotype [80]. The DcR3-mediated effector functions (or known as ‘non-decoy function’ is via crosslinking of heparin sulfate proteoglycans (HSPGs), such as syndecans and CD44v3 [81] (Fig. 1a). Interestingly, high concentrations of DcR3.Fc (10–30 μg/ml) induces DCs apoptosis by activating PKC-δ and JNK to upregulate DR5, thus recruiting Fas-associated death domain (FADD) to propagate the apoptotic signals [82]. Moreover, DcR3.Fc skews CD14^+^-monocytes to CD14^low^CD16^low^ CD68^high^CD206^high^HLA-DR^low^ M2 macrophages via epigenetic regulation [7, 83]. In addition, DcR3.Fc also enhances monocyte adhesion via activation of FAK [83]. Without the addition of exogenous M-CSF and RANK ligand (RANKL), incubation of DcR3.Fc with RAW264.7 macrophages and CD14^+^-monocytes induces cell differentiation into osteoclasts, while OPG cannot inhibit DcR3. Fc-induced osteoclast differentiation [84, 85]. This observation suggests that DcR3-mediaed effect is independent of RANKL. In addition, local injection of DcR3.Fc increases osteoclast numbers around trabecular bone in tibia with reduced bone density [85]. This observation demonstrates the powerful effect of DcR3.Fc to modulate myeloid cell differentiation and activation. However, another report shows that recombinant human DcR3.Fc (from R&D) can inhibit RANKL-induced osteoclastogenesis via downregulation of NFATc1 expression and induction of cell apoptosis [86]. The discrepancy may due to the different composition of recombinant DcR3.Fc proteins, as the DcR3.Fc (from Enzo® Life Science) does not contain a segment of peptide located in the C-terminus of human DcR3 [9].

**Fig. 2:**
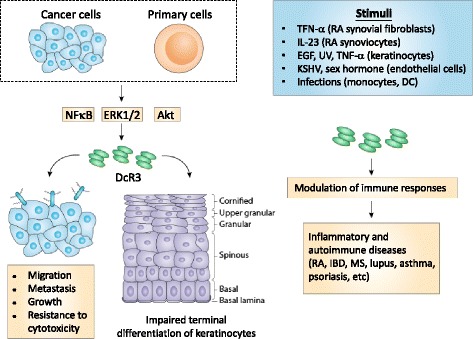
Autocrine loop of DcR3-mediated immunomodulation. Endogenous DcR3 is induced in cancer cells or keratinocytes by various stimuli, including UV, sex hormone, viruses, and cytokines. Endogenous DcR3 enhances tumor migration, metastasis, growth, and resistance to chemotoxicity in tumor cells. DcR3 is also upregulated in psoriatic lesions and impairs keratinocyte differentiation. Aberrant expression of DcR3 and DcR3 variants are observed in rheumatoid arthritis (RA), inflammatory bowel disease (IBD), multiple sclerosis (MS), systemic lupus erythematosus (SLE), and asthma
D. **Rheumatoid arthritis fibroblasts-like synoviocytes (RA-FLS):**
  We have demonstrated that DcR3.Fc is able to upregulate the expression of gene characteristics of M2-macrophages via epigenetic regulation via activating of HSPGs [7]. In addition, DcR3.Fc (from R&D) was reported to bind TL1A expressed on RA-FLS, and modulate gene expression profile. Among the 100 genes most significantly regulated by DcR3.Fc, 45 are upregulated and 55 were downregulated [89]. This observation suggests that recombinant DcR3.Fc has profound effect to modulate gene expression and cell differentiation. DcR3.Fc suppresses the expression of tryptophan hydroxylase 1, which is the rate-limiting enzyme for serotonin synthesis, in RA-FLS [90]. Furthermore, DcR3.Fc is able to induce the expression of IL12B (p40) via membrane-bound TL1A in RA-FLS [23]. It has been shown that activation of membrane-bound ligands of TNF superfamily, such as TRANCE/TNFSF11 [91] and FasL [92], can modulate cell functions by transducing ‘reverse signaling’. Thus, DcR3 may also activate its membrane ligands to modulate cell functions via triggering reverse signaling.

## Therapeutic effect of recombinant DcR3.Fc fusion protein

It has been reported that intrathecal injection of recombinant DcR3.Fc fusion protein is able to 1) ameliorate experimental autoimmune encephalomyelitis [93, 94]; 2) suppress influenza virus-induced macrophage activation and attenuate pulmonary inflammation and lethality [95]; 3) improve survival in experimental sepsis by suppressing the inflammatory response and lymphocyte apoptosis [96]; 4) improve locomotor functional recovery after spinal cord injury [9]; 5) attenuate choline-deficient (MCD) diet-induced hepatic steatosis and inflammation through its non-decoy actions to attenuate oxidative stress production [97]. All these observations suggest that DcR3.Fc is a potential therapeutic agent for the treatment of multiple sclerosis and viral pneumonia, enhance neuronal repairmen, and prevent non-alcoholic steatohepatitis.

## DcR3 mutation and human diseases

High serum level and mutations of DcR3 are concomitantly observed in patients with juvenile-onset systemic lupus erythematosus [98]. DNA sequencing identifies 2 novel missense mutations: c.C167T (p.T56I) in an adult systemic lupus erythematosus patient and c.C364T (p.H122Y) in a juvenile patient [98]. This observation suggests that defective DcR3 may contributes to the pathogenesis of systemic lupus erythematosus. It will be important to examine whether other autoimmune diseases are resulted from the DcR3 mutation of DcR3 in the future.

In addition, DcR3 mutant defective in secretion from cultured cells is greater in the Crohn’s patients [99]. Genome-wide association studies of pediatric Crohn’s disease identify common variation at the 20q13 locus, which harbors DcR3. Furthermore, a greater number of missense DcR3 variants defective in secretion from cultured cells are identified by DNA sequencing. These observations suggest that these DcR3 variants may contribute to the pathogenesis of some IBD cases, and recombinant DcR3 may be able to attenuate alimentary tract inflammation in IBD patients.

## Conclusion

It is almost 19 years since the observation that DcR3 is overexpressed in various cancer cells and is able to neutralize FasL-mediated apoptosis [2]. DcR3 apparently executes its biological functions via ‘decoy’ ability to neutralize FasL, LIGHT, and TL1A. The TL1A/DR3 axis is a novel immune pathway that participates in the pathogenesis of a variety of autoimmune rheumatic diseases, thus recombinant DcR3.Fc is a promising therapeutic agent for the treatment of inflammatory arthritis [10]. In addition, DcR3.Fc fusion protein-mediated M2-driven effect is via non-decoy functions in diseases not involving FasL, LIGHT, and TL1A. Therefore, DcR3 might have potentials for the repairing of neuronal injury and prevention of liver fibrosis. In contrast, inhibition of DcR3-mediated immunomodulatory functions seems a promising approach to attenuate tumor progression. We have shown that DcR3-mediated effect is via upregulating histone deacetylase (HDAC) [7], and addition of HDAC inhibitor is able to reduce tumor progression in the animal model [8]. Thus, DcR3 is a promising target to increase the efficacy of cancer therapy in the future.

However, the physiological functions of DcR3 are still unclear. Higher DcR3 expression was found in undifferentiated than in well-differentiated keratinocytes during tissue repair [16], and upregulation of DcR3 during skin injury is detected by microarray analysis (unpublished data). These observations suggest that DcR3 may be able to enhance repairing of skin injury. It has been shown that FasL plays a role to reduce the cellularity during skin wound healing in mice [100, 101], and LIGHT promotes collagen accumulation and skin fibrosis [102]. Thus, upregulation of DcR3 may increase cellularity by neutralizing FasL, and prevent fibrosis via neutralizing LIGHT in skin. One of the most prominent differences between human and mouse in wound healing is the presence of granulation tissue formation and re-epithelization in human, while subcutaneous muscle contraction is only observed in mouse skin [103]. Because mouse does not have DcR3, DcR3-mediated effect in human skin may be via enhancing granulation tissue formation and re-epithelization by M2 macrophages. Recently, we further find that DcR3 is able to attenuate beta-amyloid-induced neuroinflammation via skewing microglia-into M2a phenotype [104]. Thus, the physiological functions of DcR3 may be via ‘decoy’ and ‘non-decoy’ functions to create a microenvironment to favor tissue repairing.

DcR3.Fc is able to induce signaling cascades via HSPGs (syndecan-2 and CD44v3), and activate PKC → c-Src → FAK pathway to induce cell adhesion [81, 105]. DcR3-induced M2-like macrophages is also via activating HSPGs [7], and in vivo injection of HBD.Fc, which comprises a stretch of positive amino acid residues fused with Fc portion of IgG, has similar effect as DcR3.Fc to attenuate influenza virus-induced pulmonary inflammation [95]. This observation suggests that DcR3-mediated anti-inflammation is not via neutralizing effect, but via ‘non-decoy’ functions. The molecular mechanism of DcR3.Fc-mediated anti-inflammation needs to be further studied in the future.
